# Biomechanics and early sac regression after endovascular aneurysm repair of abdominal aortic aneurysm

**DOI:** 10.1016/j.jvssci.2023.100104

**Published:** 2023-03-30

**Authors:** Marko Bogdanovic, Antti Siika, Moritz Lindquist Liljeqvist, T. Christian Gasser, Rebecka Hultgren, Joy Roy

**Affiliations:** a Department of Molecular Medicine and Surgery, Karolinska Institutet, Stockholm, Sweden; b Department of Vascular surgery, Karolinska University Hospital, Stockholm, Sweden; c Department of Engineering Mechanics, KTH Royal Institute of Technology, Stockholm, Sweden; d Faculty of Health Sciences, University of Southern Denmark, Odens, Denmark

**Keywords:** EVAR, AAA, Sac regression, Sac change, Biomechanical analysis, ILT

## Abstract

**Background:**

Sac regression after endovascular aneurysm repair (EVAR) of abdominal aortic aneurysms (AAA) is regarded as a marker of successful response to treatment. Several factors influence sac behavior after EVAR, yet little is known about the value of preoperative biomechanics. The aim of this study was to investigate the difference in aortic biomechanics between patients with and without sac regression.

**Methods:**

Patients treated with standard EVAR for infrarenal AAA at the Karolinska University Hospital between 2009 and 2012 with one preoperative and a minimum of two postoperative computed tomography angiography (CTA) scans were considered for inclusion in this single-center retrospective cohort study. Biomechanical indices such as AAA wall stress and wall stress-strength ratio as well as intraluminal thrombus (ILT) thickness and stress were measured preoperatively in A4ClinicRE (VASCOPS GmbH). AAA diameter and volume were analyzed on preoperative, 30-day, and 1-year CTAs. Patients were dichotomized based on sac regression, defined as a ≥5 mm decrease in maximal AAA diameter between the first two postoperative CTA scans. Multivariable logistic regression was used for analysis of factors associated with early sac regression.

**Results:**

Of the 101 patients treated during the inclusion period, 64 were included. Thirty-nine (61%) demonstrated sac regression and 25 (39%) had a stable sac or sac increase. The mean patients age (73 years vs 76 years), male sex (85% vs 96%), and median AAA diameter (58 mm vs 58.5 mm) did not differ between patients with and without sac regression. Although no difference in preoperative biomechanics was seen between the groups, multivariable logistic regression revealed that a larger AAA diameter (odds ratio [OR], 1.27; 95% confidence interval [CI], 1.06-1.51; *P* = .009) and smoking (OR, 22.1; 95% CI, 2.78-174; *P* = .003) were positively associated with sac regression. In contrast, the lumen diameter (OR, 0.87; 95% CI, 0.77-0.98; *P* = .023), ILT thickness (OR, 0.85; 95% CI, 0.75-0.97; *P* = .013), aspirin or direct-acting oral anticoagulant use (OR, 0.11; 95% CI, 0.02-0.61; *P* = .012), and mean ILT stress (OR, 0.35; 95% CI, 0.14-0.87; *P* = .024) showed a negative association. Patients with sac regression had fewer reinterventions (log-rank *P* = .010) and lower mortality (log-rank *P* = .012) at the 5-year follow-up.

**Conclusions:**

This study, characterizing preoperative biomechanics in patients with and without sac regression, demonstrated a negative association between mean ILT stress and ILT thickness with a change in sac diameter after EVAR. Given that the ILT is a highly dynamic entity, further studies focusing on the role of the thrombus are needed. Furthermore, patients presenting with early sac regression had improved outcomes after EVAR.

**Article Highlights**

• **Type of Research:** Single-center, case control study with retrospective analysis of prospectively collected registry data from the Stockholm Abdominal Aortic Aneurysm Biobank
• **Key Findings:** Significant sac regression within the first postoperative year was seen in 39 patients (61%) treated with standard endovascular aneurysm repair (EVAR) for infrarenal abdominal aortic aneurysm. The mean intraluminal thrombus (ILT) stress and ILT thickness derived by biomechanical analysis of patient-specific computed tomography angiography was negatively associated with sac change after EVAR in a multivariable logistic regression model.
• **Take Home Message:** Preoperative biomechanical analysis of the ILT may aid in predicting sac change after EVAR.

Endovascular aneurysm repair (EVAR) has surpassed open surgical repair (OSR) as the primary treatment for infrarenal abdominal aortic aneurysm (AAA) in most Western vascular surgery centres.^1^, ^2^, ^3^ Albeit superior to OSR in short-term outcomes, EVAR is associated with a greater need for reintervention, as well as inferior long-term survival.^4^^,^^5^ Several large randomized, controlled trials have observed an immediate survival benefit of EVAR over OSR, which diminishes after the first year and is equalized after 3 years.^6^, ^7^, ^8^, ^9^ Endograft-related complications such as endoleaks, stent migration, and limb graft occlusion collectively constitute the major cause of reintervention and rupture after EVAR.^5^^,^^10^^,^^11^ Endoleaks of any type are seen in up to one-half of all EVAR patients.^12^ Distinguishing which EVAR patient will do well and, conversely, which will require a more rigorous follow-up protocol with additional procedures is a major challenge in the endovascular era and needs continued efforts from the vascular surgery community.^1^^,^^13^

Sac regression, defined in reporting standards as a ≥5 mm decrease in AAA diameter is seen in up to one-half of all EVAR patients.^14^, ^15^, ^16^, ^17^, ^18^ Contemporary data from several groups suggest that early sac regression, within the first postoperative year, may act as a surrogate marker for EVAR success and is associated with improved surgical outcomes as well as long-term survival.^16^^,^^17^^,^^19^^,^^20^ Certain factors have been proposed to influence sac regression both positively (AAA size, statin therapy, chronic kidney disease) and negatively (age, endoleak presence, thrombus burden), yet results vary between studies. Interestingly, smoking has been associated with a greater degree of sac change, and current smokers have a lower risk of expansion after EVAR.^21^, ^22^, ^23^ Biomechanical assessment with finite element analysis (FEA) of AAAs has been used extensively in studying rupture risk.^24^^,^^25^ Today’s commercially available software uses patient-specific imaging to calculate local and overall biomechanical stress acting on the aneurysm tissues. There is, however, limited knowledge on its use and value for characterizing AAAs that will respond well to EVAR and, furthermore, its potential in prediction of sac change after AAA treatment.

The primary aim of this study was to assess if preoperative biomechanics and morphology are associated with early sac regression after EVAR. The secondary aim was to investigate predictive factors of sac regression. The third aim was to explore whether aneurysm volume change is a more comprehensive indicator of complications after EVAR than change in AAA diameter.

## Methods

### Study cohort

Between January 2009 and June 2012, all patients undergoing EVAR for AAA at the Vascular Surgery Department of Karolinska University Hospital were identified and considered for inclusion in this retrospective observational cohort study. Nonstandard EVAR procedures (thoracoabdominal aneurysms or suprarenal or juxtarenal AAAs) and nondegenerative AAAs (mycotic, genetic, trauma induced) were exempted from assessment. Further inclusion criteria were preoperative contrast enhanced computed tomography angiography (CTA) within 6 months from the index surgery and a minimum of two postoperative follow-up contrast-enhanced CTAs (the first approximately 30 days after surgery and the second within 6-18 months from EVAR, as per institution protocol). Relevant clinical and operative variables were extracted from the electronic medical records and local surgery planning system.

Sac regression was defined as a ≥5 mm/year maximal diameter decrease measured between the first and second postoperative follow-up CTA in accordance with published reporting standards.^18^ Cases with sac regression between the follow-up CTAs were labeled “responders” and conversely, subjects with sac increase or no change were termed “nonresponders.” Secondary outcomes included comparing preoperative biomechanical characteristics in EVAR patients, as well as survival and freedom from reintervention by responder status. Participants could accrue follow-up time from the date of primary intervention to date of death, loss to follow-up, or March 31, 2021. All-cause as well as AAA-related mortality were recorded, along with endograft-specific outcomes. The reporting of this study is consistent with the Strengthening the Reporting of Observational studies in Epidemiology statement and was approved by the Swedish Ethical Review Authority. All clinical and patient-related data as well as CTA images were anonymized at collection.

### Biomechanical and morphological analyses

FEA was performed on all preoperative CTAs using A4 ClinicsRE 5.0 (VASCOPS, GmbH, Graz, Austria). The process includes three-dimensional reconstruction of the AAA with semiautomatic differentiation of the tissue types including the vessel wall, lumen and intraluminal thrombus (ILT). The three-dimensional model is subsequently meshed and simulations are run with both neutral and patient-specific characteristics. In the former analysis, all patients were given a mean arterial pressure of 100 mm Hg, negative heredity for AAA, male sex, and age of 65 years. A4 ClinicsRE 5.0 considers the AAA wall and ILT incompressible, hyperelastic, and isotropic based on previously published modelling.^26^, ^27^, ^28^, ^29^, ^30^ FEA yields several biomechanical variables including peak wall stress, the maximum stress in the AAA wall; the peak wall rupture index, which is the largest ratio between wall stress and strength; the mean estimated ILT stress as well as ILT thickness and volume; and the lumen diameter and aneurysm volume.

All available postoperative CTAs were reviewed for measurement of maximal AAA diameter (dMax) and volume, using 3Mensio Vascular software (Pie Medical Imaging, Maastricht, the Netherlands). Volume analysis was adapted from the St George's Vascular Institute protocol.^31^ All dMax measurements were performed in multiplanar reconstruction mode, outer to outer wall orthogonally to the AAA centerline according to previously published reporting standards.^18^ Features of the inferior mesenteric and lumbar arteries (diameter and patency) were assessed on axial images and diameter was measured across the vessel width perpendicular to the flow as in standard institutional practice.

### Statistical analyses

Baseline continuous values are displayed as mean and standard deviation or median and interquartile range. Categorical values are presented as count and percentage. Normality of the data was tested with the Shapiro-Wilk test. Continuous and categorical data were, depending on distribution and expected sample size, tested with the Student *t* test or Mann-Whitney *U* test and χ^2^ or Fisher's exact test, respectively. Owing to heterogeneity in the time interval between the follow-up CTAs used for calculating sac change, the measurements were normalized with the following formula:

$$\frac{dMax\;change\;between\;first\;and\;second\;follow-up\;CTA\;\left(mm\right)}{Time\;between\;the\;first\;and\;second\;follow-up\;CTA\;\left(months\right)}\times 12$$

Pearson correlation was used to correlate change in dMax with change in AAA volume within the first year post-EVAR. Univariable and multivariable logistic regression analyses with backward stepwise conditional selection were used for analysis of potential predictors of early sac regression. The backward selection method was utilized in order to minimize overfitting of the prediction model. Receiver operating characteristic curve with the area under curve as test was used for prediction of endograft-related complications from dMax and AAA volume change during the first postoperative year, respectively. Kaplan-Meier curves were used to visualize survival and freedom from reintervention, and log-rank constituted significance testing between the groups. Any null hypothesis was rejected if the two-sided *P* value was <.05. All statistical analysis was performed with SPSS Statistics version 27.0 (IBM Corp, Armonk, NY).

## Results

### Study cohort characteristics

**Fig 1:**
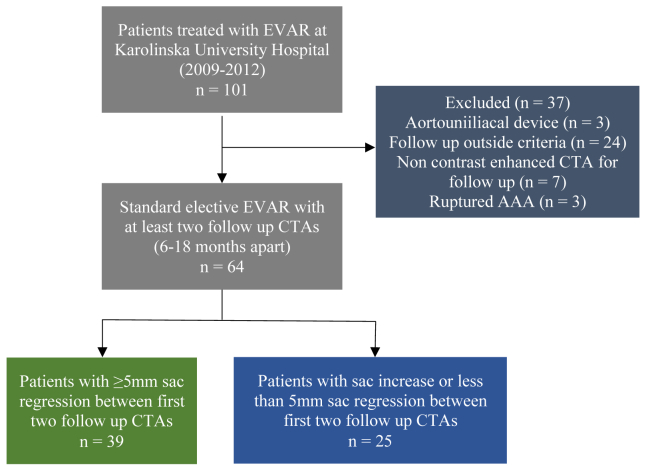
Flow chart of study design and inclusion of patients. *AAA,* Abdominal aortic aneurysm; *CTA*, computed tomography angiography; *EVAR*, endovascular aneurysm repair.

Of the 101 patients who underwent EVAR between 2009 and 2012, a total of 64 patients were included in the study. See the flowchart in Fig 1 for details. The study cohort was categorized into a responder (patients with a ≥5 mm sac regression between the 30-day and 1-year follow-up CTA normalized by moths, n = 39) and nonresponder group (patients with sac increase or sac regression of <5 mm, n = 25). The distribution of baseline characteristics for the two groups is displayed in Table I. No difference in age at time of EVAR was seen for the responder compared with the nonresponder group (73.2 years vs 75.9 years; *P* = .168). The distribution of sex was similar in both groups. No difference in median AAA size and volume at 30 days after EVAR was seen between the groups. However, responders had a longer median follow-up compared with nonresponders (115.2 months vs 101.8 months; *P* = .007) and had more frequently a history of smoking (current or former, 92.3% vs 68%; *P* = .018). There were no differences between the two groups in regards to comorbidities, medications, or endograft device type used (Table I). Features of the inferior mesenteric and lumbar arteries such as patency and diameter did not differ.

**Table I:** Baseline characteristics of 64 EVAR patients, by responder status

Characteristics	Responder (n = 39)	Nonresponder (n = 25)	*P* value
Age, years	73.2 ± 6.9	75.9 ± 8.9	.168
Male	33 (84.6)	24 (96)	.231
Median dMax, mm (IQR)	58 (55-67)	58.5 (54-67)	.741
Median volume, cm^3^ (IQR)	190 (152.4-259)	201 (152-258)	.715
Follow-up time, months	115.2 (32-143)	101.8 (16-133)	.007
Smoking status (ever vs never)	36 (92.3)	17 (68)	.018
Smoking status			
Never	4 (10.3)	8 (32)	.047
Former	18 (41)	13 (52)	.798
Current	19 (48.7)	4 (16)	.008
Comorbidities			
Hypertension	30 (76.9)	16 (64)	.393
AMI	11 (28.2)	6 (24)	.778
Angina	6 (15.4)	8 (32)	.134
Lower PAD	4 (10.3)	4 (16)	.701
Diabetes type 2	5 (12.8)	1 (4)	.391
Stroke	2 (5.1)	2 (8)	.640
AAA heredity	4 (10.3)	3 (12)	.999
Medications			
ASA/clopidogrel	22 (56.4)	20 (80)	.064
DOAC/warfarin	7 (17.9)	2 (8)	.463
Statin	24 (61.5)	19 (76)	.282
Morphological features			
Lumbar arteries open	41 (12.5)	25 (12.5)	.233
Lumbar artery diameter >2 mm	7 (2.2)	6 (3)	.961
IMA diameter >2.5 mm	6 (15.3)	7 (28)	.665
IMA patency	18 (46)	16 (64)	.228
Endograft type			
Zenith Flex	15 (38.5)	8 (32)	.596
Zenith LP	0 (0)	1 (4)	na
Medtronic Endurant	20 (51.3)	13 (52)	.999
Gore Excluder	4 (10.3)	3 (12)	.565

*AAA*, Abdominal aortic aneurysm; *AMI*, acute myocardial infarction; *ASA*, acetylsalicylic acid; *dMax*, maximal diameter at first postoperative computed tomography angiography scan; *DOAC*, direct-acting oral anticoagulant; *EVAR*, endovascular aneurysm repair; *IMA*, internal mesenteric artery; *IQR*, interquartile range; *LP*, low profile; *na*, not applicable; *PAD*, peripheral arterial disease.

Values are expressed as mean ± standard deviation, number (%), or median (min-max) unless stated otherwise.

Responder denotes patients with >5 mm sac regression between first and 1-year postoperative computed tomography angiography (CTA). Volume refers to measurement on first CTA postoperatively.

### Postoperative complications and imaging details

Endograft-related complications and imaging details by responder status are presented in Table II. Patients were stratified according to dMax shrinkage between the first and second postoperative CTAs; consequently, responders had a larger regression of dMax and AAA volume (−13.8 mm/year vs −0.45 mm/year and −68.9 cm^3^ vs −6.4 cm^3^, respectively; *P* < .001 for both comparisons). A majority of the nonresponders suffered endograft-related complications (of any type), as well as more endoleak complications compared with responders (84% vs 41% and 84% vs 19.5%, respectively; *P* < .001 for both comparisons). There were no significant differences in the time interval between the preoperative CTA and index EVAR or months between the postoperative CTAs used for assessment of sac regression for the two groups.

**Table II:** Complications after endovascular aneurysm repair (*EVAR*) and imaging specifications of 64 EVAR patients, by responder status

Complications	Responder (n = 39)	Nonresponder (n = 25)	*P* value
Patients with endograft-related complication during follow-up	16 (41)	21 (84)	<.001
Patients with any endoleak type during follow-up	8 (19.5)	20 (80)	<.001
Type Ia	2 (5.1)	3 (12)	
Type Ib	0 (0)	4 (16)	
Type II	4 (10.3)	13 (52)	
Type III	0 (0)	0 (0)	
Stenosis	5 (13)	2 (7.5)	
LGO	4 (10.3)	0 (0)	
Rupture	1 (2.6)	0 (0)	
Patients who had reinterventions	10 (25.5)	11 (44)	.111
Patients with any EVAR-related complication (within the first postoperative year	4 (9.7)	13 (52)	<.001
Imaging			
Months between preoperative CTA to operative	3.9 (0.2 to 9.5)	2.6 (0.2 to 8.9)	.250
Months between 2 scans used for assessment of sac dynamics, median (min-max)	11.8 (6 to 18)	11.7 (6 to 18)	.736
Mean change dMax (1 mo to 1 y), mm (min-max)	−13.3 (−28 to −5.0)	−0.41 (−4.7 to 5.7)	<.001
Mean change volume (mo to 1 y), cm^3^ (min-max)	−67.71 (−147 to −17)	−6.4 (−83 to 48)	<.001

*CTA*, Computed tomography angiography; *dMax*, maximal diameter at first postoperative computed tomography angiography scan; *LGO*, limb graft occlusion; *nonresponder*, <5 mm sac regression between first 2 postoperative scans; *responder*, >5 mm sac regression between first 2 postoperative scans.

Values are expressed as number (%) or median (min-max) unless stated otherwise.

Responder denotes patients with >5 mm sac regression between first and 1-year postoperative CTA. dMax and volume change refers to measurements on first and 1-year postoperative CTA.

### Preoperative biomechanical characteristics

FEA analysis run with patient-specific characteristics on preoperative CTAs presented in Table III by responder status showed a trend toward a lower mean ILT stress in the responder group compared with the nonresponders (7.2 kPa vs 7.6 kPa; *P* = .074). No other biomechanical parameters differed between the groups. Repeating the same simulations with neutral patient characteristics (specified in the Methods) yielded similar results.

**Table III:** Preoperative biomechanical characteristics of 66 endovascular aneurysm repair (EVAR) patients

Biomechanical characteristics (preoperative)	Responder (n = 39)	Nonresponder (n = 25)	*P* value
PWS, kPa	218.6 ± 56.7	237.6 ± 51.6	.248
PWRI, ratio	0.48 ± 0.13	0.52 ± 0.29	.315
Lumen diameter, mm	44.8 ± 11	45 ± 9.5	.989
Lumen volume, cm^3^	101 ± 52.5	95.2 ± 48.6	.587
ILT thickness, mm	21.3 ± 10.5	24.1 ± 12.9	.339
ILT volume, cm^3^	86.4 ± 57.2	94.5 ± 65.4	.907
Mean ILT stress, kPa	7.2 ± 0.77	7.6 ± 0.98	.074
Maximum ILT stress, kPa	23.8 ± 6.9	29.2 ± 16.4	.184

*ILT*, Intraluminal thrombus; *PWS*, peak wall stress; *PWRI*, peak wall rupture index.

Values are expressed as mean ± standard deviation.

### Potential factors affecting early sac regression

Univariable and multivariable logistic regression analyses were used to examine potential variables affecting responder status (significant sac change between 1 month and 1 year of follow-up) (Table IV). In the univariable analysis, smoking status was positively associated with early sac regression (odds ratio [OR], 5.64; 95% confidence interval [CI], 1.32-23.9; *P* = .019). Conversely, aspirin/direct-acting oral anticoagulant use (OR, 0.32; 95% CI, 0.10-1.04; *P* = .058) and mean ILT stress (OR, 0.58; 95% CI, 0.31-1.06; *P* = .078) showed a trend of negative association with early sac regression.

**Table IV:** Uni- and multivariable logistic regression with potential predictors of responder status

Potential predictors	Uni- variable	95% CI	*P* value	Model 1^a^	95% CI	*P* value
	OR			OR		
Age	0.95	(0.89-1.02)	.167			
Gender	0.23	(0.02-2.03)	.186			
dMax at 1 mo postoperative	1.00	(0.95-1.06)	.917	1.27	(1.06-1.51)	.009
PWS	0.99	(0.98-0.98)	.283			
PWRI	0.42	(0.04-4.79)	.486			
Lumen diameter	0.99	(0.95-1.05)	.973	0.87	(0.77-0.98)	.023
Lumen volume	1.00	(0.99-1.01)	.618			
ILT thickness	0.98	(0.93-1.02)	.334	0.85	(0.75-0.97)	.013
ILT volume	0.99	(0.99-1.00)	.756			
Mean ILT stress	0.58	(0.31-1.06)	.078	0.35	(0.14-0.87)	.024
Ever vs never smoker	5.64	(1.32-23.9)	.019	22.1	(2.78-174)	.003
Aspirin/DOAC use	0.32	(0.10-1.04)	.058	0.11	(0.02-0.61)	.012

*DOAC*, Direct-acting oral anticoagulant; *dMax*, maximal diameter; *ILT*, intraluminal thrombus; *PWS*, peak wall stress; *PWRI*, peak wall rupture index; *95% CI*, 95% confidence interval.

a Model 1 is a backward stepwise (conditional) selection model adjusting for all variables from the univariate analysis producing a finite model to minimize overfitting.

In the multivariable model, backward stepwise conditional selection was performed by adjusting for all variables from the univariable analysis in order to find the most optimal regression model. A larger dMax at 1 month postoperatively (OR, 1.27; 95% CI, 1.06-1.51; *P* = .009) and smoking status (OR, 22.1; 95% CI, 2.78-174; *P* = .003) were associated with sac regression. In contrast, lumen diameter (OR, 0.87; 95% CI, 0.77-0.98; *P* = .023), ILT thickness (OR, 0.85; 95% CI, 0.75-0.97; *P* = .013), aspirin/direct-acting oral anticoagulant use (OR, 0.11; 95% CI, 0.02-0.61; *P* = .012), and mean ILT stress (OR, 0.35; 95% CI, 0.14-0.87; *P* = .024) were associated negatively with early sac regression.

### Prediction of endograft-related complications

**Fig 2:**
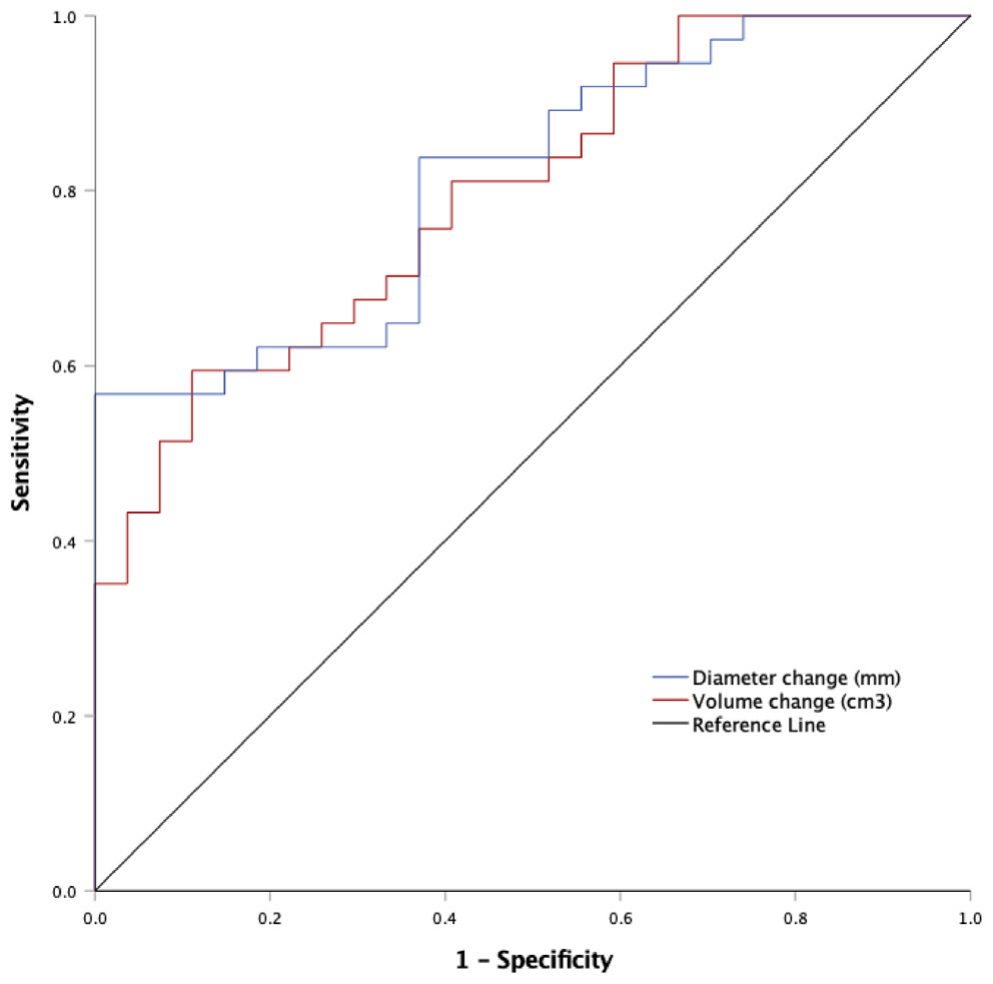
Receiver operating characteristic curve of diameter and volume change predicting endograft-related complications after endovascular aneurysm repair (EVAR). Prediction of endograft-related complications during the total follow-up time by early (within the first year) diameter and volume change after EVAR. Area under the curve (AUC) for diameter change = 0.813; *P* < .001, and for volume change, AUC = 0.797, *P* < .001.

AAA dMax and volume change within the first postoperative year were used as classifiers for predicting whether a patient would suffer any endograft-related complication during follow-up and is presented with a receiver operating characteristic curve (Fig 2). The area under curve for dMax and volume change was 0.813 and 0.797, respectively (*P* <.001 for both variables). At the 92% sensitivity level, with 44% specificity, a sac regression of ≥15 mm at dMax within the first postoperative year could predict complete freedom from endograft complications, which represented one-fourth (25%) of the patients. Similarly, a volume decrease of ≥82 cm^3^ could predict freedom from endograft-related complications with 92% sensitivity and 41% specificity, representing close to one-fourth (23%) of the patients.

### Survival and reintervention

**Fig 3:**
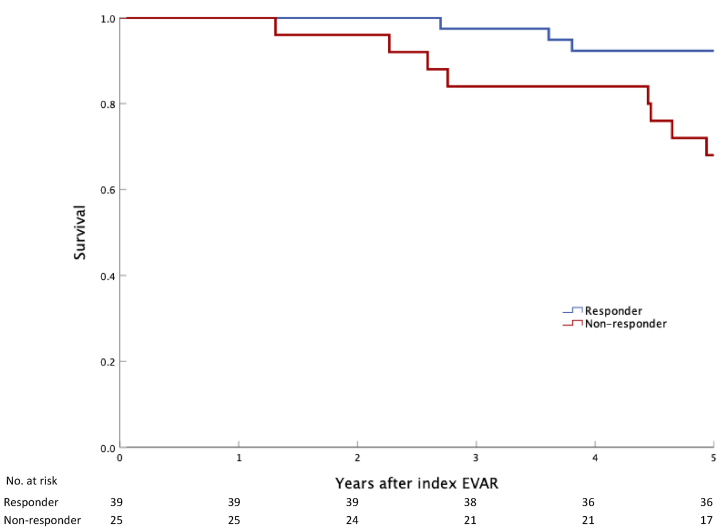
Survival at 5 years after endovascular aneurysm repair (*EVAR*) by responder status. Kaplan-Meier plot of survival during 5-year follow-up after EVAR, stratified by responder status (>5 mm sac regression between first and second postoperative computed tomograph angiography [CTA]). *P* = .012 (log-rank test).

**Fig 4:**
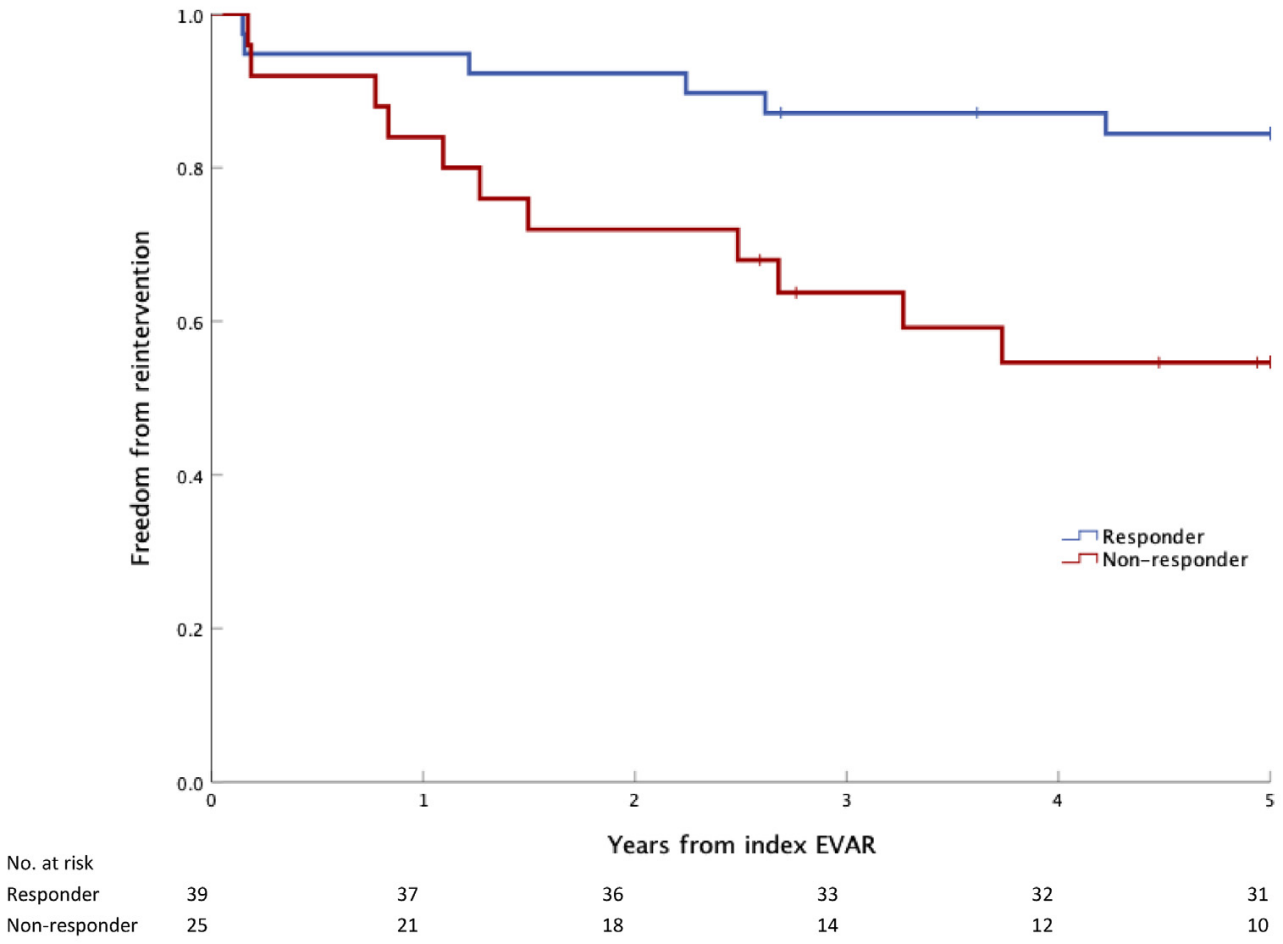
Freedom from all vascular reinterventions at 5 years after endovascular aneurysm repair (*EVAR*) by responder status. Kaplan-Meier plot of freedom from reintervention during the 5-year follow-up after EVAR, stratified by responder status (>5-mm sac regression between the first and second postoperative computed tomography angiography scans [CTAs]). *P* = .010 (log-rank test).

The 5-year all-cause mortality was lower in the responder group compared to the nonresponder group (log-rank *P* = .009). Only three responders (7.3%) compared with eight nonresponders (32%) passed away during the initial 5-year period, as presented with a Kaplan-Meier plot in Fig 3. Similarly, reinterventions at five years after EVAR were lower for the responder group in contrast with the nonresponders (Fig 4) (*P* = .006). The median survival time for the responder group was 9.5 years compared with 8.3 years for nonresponders (*P* = .048).

## Discussion

This cohort study of patients with and without early sac regression suggests that ILT thickness and mean ILT stress, two biomechanical variables previously unstudied in the context of sac regression, are associated with sac change. However, no difference in aneurysm wall biomechanical indices were seen between the groups. This study is, to our knowledge, the first to describe preoperative biomechanics in AAA and the association with sac change after EVAR. The dMax and volume change between the first and second follow-up CTA could predict freedom from endograft complications after EVAR, with similar distinction. Although a crude measurement, the significance of early sac regression as a surrogate for successful response to EVAR is becoming increasingly evident and may have an impact on postoperative surveillance protocols.^1^^,^^13^ In similarity with previous studies, patients with a ≥5 mm decrease in AAA maximal diameter after EVAR demonstrated fewer endograft complications and reinterventions at 5 years as well as longer median survival time compared with nonresponders.^16^^,^^17^^,^^22^^,^^23^^,^^32^

### Preoperative aortic morphology and biomechanics in EVAR patients

In our study, no differences were seen in internal mesenteric artery patency and size, as well as number of lumbar arteries between the groups. These factors have been associated negatively with sac regression in previous publications.^33^^,^^34^ Clinical observational studies suggest that a minority of type II endoleaks lead to reinterventions.^35^^,^^36^ Biomechanical analysis of AAA growth and rupture risk has been investigated previously, linking aneurysm-related events with the biomechanical properties of the vessel wall and ILT.^37^ ILT stress (force divided by area) is the stress acting upon the thrombus itself and is based on ex vivo tensile testing of ILT material. ILT stress was measured by the commercially available software A4 ClinicsRE 5.0 and considers the tissue to be isotropic, hyperelastic, and porous. Although still experimental, the method is reproducible, with adequate interobserver and intraobserver variability, and has been validated in previous publications.^28^^,^^29^^,^^38^ ILT stress has been correlated with the biological expression of both d-dimer and neutrophil elastase-derived cross-linked fibrin degradation products.^39^^,^^40^ Although there were no significant differences in wall-related biomechanical variables (peak wall stress, peak wall rupture index) between the responder vs nonresponder groups, logistic regression analyses suggest there are characteristics of the ILT (mean ILT stress and thickness) negatively associated with early sac change after EVAR. These data support the notion of the ILT being a biologically active tissue, and any change in the sac size is directly related to changes in the thrombus of the aneurysmal sac. Under normal conditions, the ILT offers the underlying vessel wall some support, regardless of its porous structure that enables blood pressure to propagate through it.^41^ However, under increased stress in the ILT, small contained fractures may develop, providing additional compartments for proteolytic activity.^42^, ^43^, ^44^ High ILT stress likely promotes AAA growth preoperatively, which could perhaps explain why it was found influential in this study. There is however, to our knowledge no literature on how ILT stress changes after an endograft has been implanted and why (as per our results) higher ILT stress would impede sac regression. Different aspects of thrombus burden (size, volume) and localization have been studied preoperatively in regards to post-EVAR outcomes and sac change with conflicting results.^45^, ^46^, ^47^, ^48^ Biomechanical characteristics of the ILT may therefore be of importance in relation to sac change after EVAR.

### Predictors of sac regression

Previously studied factors affecting sac change after EVAR include age, anticoagulation, smoking, AAA size, statin therapy, diabetes mellitus, renal insufficiency, and endoleaks, yet some of the literature is contradictory.^21^ In the current study, aspirin use was associated with less early sac regression, whereas age showed no effect. Lalys et al^21^ recently published a comprehensive systematic review on factors affecting sac regression after EVAR. Out of the twelve studies reporting age as a risk factor, half did not demonstrate a significant association with sac change. Owing to high heterogeneity for this outcome, age was not included in the meta-analysis. The association between high age and inferior outcomes after EVAR is however uncontroversial.^1^

Regarding anticoagulation, Aoki et al^49^ investigated the effect of tranexamic acid (TXA) on sac change after EVAR and found greater sac regression in patients administered TXA daily at 6 months follow-up compared with patients not treated with TXA. The rationale is that antifibrinolytic therapy enables the complete occlusion of visceral vessels after endograft implantation, inhibiting endoleak formation and potentiating sac regression. European guidelines recommend all patients with AAA be on low-dose aspirin, a drug that counteracts primary hemostasis. In the current study, aspirin use was lower in the responder group compared with nonresponders at time of the index EVAR. Furthermore, in the multivariable analysis, aspirin use was negatively associated with sac regression. The number of patients on direct acting oral anticoagulants or warfarin were very few and did not differ between the groups. However, previous studies suggest an increased incidence of type II endoleaks and reintervention in patients treated with warfarin compared with antiplatelet therapy.^50^ Similarly, Biebl et al^51^ noticed a higher incidence of early endoleaks in patients on warfarin compared with no anticoagulation, but there was no effect on the reintervention rate or survival.

Similar to the influence of age, maximal preoperative diameter as a factor for sac change is inconclusive, and reviewing the effect of continuous factors is challenging owing to heterogeneity between studies.^21^ This study used the maximal diameter from the 30-day follow-up CTA in an attempt to minimize uncertainty of aneurysm growth during the time to EVAR. The data suggest that a larger dMax at 30 days was associated with greater sac regression, recently corroborated by van Rijswijk et al.^15^ Previous studies may introduce bias by using the preoperative maximal diameter and hence alter the results. Furthermore, this study suggests that current or previous smoking increases sac regression, a finding supported by others.^52^^,^^53^ Endoleak incidence is lower in smokers and one theory implies that it is due to the prothrombogenic effects of tobacco.^21^ Certainly, the current results do not justify continuation or initiation of smoking nor termination of antiplatelet therapy or anticoagulation after EVAR. These topics warrant further studies.

### Diameter and volume change after EVAR

Several groups have highlighted AAA volume as a more precise and comprehensive variable to use in postoperative follow-up after EVAR, compared with measurements of dMax.^32^^,^^54^^,^^55^ Stable dMax after EVAR may be caused by an undetected endoleak, which is where volume measurement perhaps plays a role. Clear consensus on a specific threshold of volume change to define sac regression is, however, still missing, and it has yet to become a part of postoperative follow-up protocols.^32^^,^^55^, ^56^, ^57^ In this study, we show that cut-offs of ≥15 mm for reduction in dMax and ≥82 cm3 in volume could predict freedom from endograft-related complications with a sensitivity of >90%. In contrast, Franchin et al^32^ demonstrated that the absence of volume shrinkage correlated more strongly with unfavorable results after EVAR than diameter change.

### Sac regression and EVAR outcomes

This study suggests that early sac regression is associated with significant freedom from reintervention, as well as greater overall survival. Although this notion has been investigated in previous publications, sac regression is yet to be incorporated in societal follow-up protocols after EVAR.^1^^,^^13^ Current Society for Vascular Surgery guidelines consider sac enlargement as a hostile marker requiring more frequent postoperative diagnostics, yet there are indications that even stable sacs without presence of endoleaks may be of importance and should not be neglected.^13^^,^^58^ Regarding early sac regression, Bastos Goncalves et al^16^ reported that patients with a ≥10-mm sac decrease had significantly fewer reinterventions compared with stable and moderately decreasing (5-9 mm) aneurysm sacs. Cieri et al^19^ presented a large cohort of EVAR cases with improved long-term survival (≤10 years) in patients with >5 mm of sac regression. Similarly, Houballah et al^20^ demonstrated a significant association between sac regression and fewer endograft-related complications after EVAR. Both mortality and rate of fatal events seem to be fewer in patients with sac regression, as seen in two more recent publications.^58^^,^^59^ Collectively, the literature seems to agree that significant sac regression is a robust indicator of successful EVAR. In their systematic review and meta-analysis of the prognostic value of sac regression, Antoniou et al^17^ went further, proposing a follow-up algorithm with assessment of sac change at the 1 year after EVAR by ultrasound examination.

### Limitations

The present study possesses inherent limitations related to its retrospective design, including the risks of missing data and selection bias. The prospectively collected biobank used for this investigation is, however, consecutive and has been validated internally on a regular basis since its creation in 2009. A minimum of two CTAs after EVAR were required for inclusion in the study. This criterion did not introduce selection bias because the majority of excluded patients had other reasons for exclusion than insufficient follow-up imaging. In contrast with previous publications, the time interval for sac change was normalized to 12 months, decreasing the risk of misclassification.^32^^,^^60^ Furthermore, the 30-day postoperative CTA was used as an index image for measuring sac change instead of the preoperative CTA. This decision was an effort to minimize bias from uncertainty introduced by the heterogeneous time interval and unknown AAA growth between the preoperative imaging and EVAR. Cohort stratification was, however, based on absolute (not proportional) sac change, which may result in an overestimation vs underestimation of change for large and small AAAs, respectively. A comparison between these two stratification methods was performed (not presented in this article) without showing significant differences. Although the current sample size is limited and restricts generalizability, several key outcomes in the current study are concomitant with published data on larger samples and provides reassurance as such.^17^ However, because the total number of females in this study was seven (responder n = 6, nonresponder n = 1), it was statistically not feasible to perform disaggregated analysis to investigate sex- and gender-specific outcomes. The effects of sex and gender on aneurysmal disease is an important topic that warrants additional exposure in larger cohorts. Furthermore, it is difficult to draw strong conclusions about smoking because the wide confidence interval for the predictor smoking status in the logistic regression model is probably driven by the modest sample size for a multivariable analysis.

## Conclusions

A majority of patients undergoing standard EVAR demonstrated early sac regression (≥5 mm within the first postoperative year). The importance of early sac regression for overall EVAR success is becoming increasingly evident and in the current study, these patients presented with significantly fewer endograft-related complications and reinterventions, as well as longer median survival. Aneurysm volume could predict freedom from endograft complications with equal distinction as aneurysm diameter. Although no difference was seen in terms of preoperative biomechanics of the aneurysm wall between patients with and without early sac regression, a novel finding implicating features of the ILT (mean stress and maximal thickness) were negatively associated with early sac change. Aneurysm biomechanics has a potential in predicting post-EVAR changes. However, larger studies are needed to further elucidate the role of the ILT and in particular, the implications of ILT stress and thrombus morphology on outcomes after EVAR.

## Author Contributions

Conception and design: MB, AS, MLL, TCG, RH, JR

Analysis and interpretation: MB, AS, MLL, TCG, RH, JR

Data collection: MB

Writing the article: MB, AS, MLL, TCG, RH, JR

Critical revision of the article: MB, AS, MLL, TCG, RH, JR

Final approval of the article: MB, AS, MLL, TCG, RH, JR

Statistical analysis: MB

Obtained funding: JR

Overall responsibility: JR
